# Five levels of performance and two subscales identified in the computer-vision symptom scale (CVSS17) by Rasch, factor, and discriminant analysis

**DOI:** 10.1371/journal.pone.0202173

**Published:** 2018-08-28

**Authors:** Mariano González-Pérez, Rosario Susi, Ana Barrio, Beatriz Antona

**Affiliations:** 1 Faculty of Optics and Optometry, Universidad Complutense de Madrid, Madrid, Spain; 2 Faculty of Statistical Studies, Universidad Complutense de Madrid, Madrid, Spain; Universidade do Minho, PORTUGAL

## Abstract

**Purpose:**

To quantify the levels of performance (symptom severity) of the computer-vision symptom scale (CVSS17), confirm its bifactorial structure as detected in an exploratory factor analysis, and validate its factors as subscales.

**Methods:**

By partial credit model (PCM), we estimated CVSS17 measures and the standard error for every possible raw score, and used these data to determine the number of different performance levels in the CVSS17. In addition, through discriminant analysis, we checked that the scale's two main factors could classify subjects according to these determined levels of performance. Finally, a separate Rasch analysis was performed for each CVSS17 factor to assess their measurement properties when used as isolated scales.

**Results:**

We identified 5.8 different levels of performance. Discriminant functions obtained from sample data indicated that the scale's main factors correctly classified 98.4% of the cases. The main factors: Internal symptom factor (ISF) and external symptom factor (ESF) showed good measurement properties and can be considered as subscales.

**Conclusion:**

CVSS17 scores defined five different levels of performance. In addition, two main factors (ESF and ISF) were identified and these confirmed by discriminant analysis. These subscales served to assess either the visual or the ocular symptoms attributable to computer use.

## Introduction

Many of today’s jobs involve prolonged computer use. This increases a worker's visual demands and may give rise to an array of computer-related visual and ocular symptoms (CRVOS) that adversely affect both quality of life[1] and productivity[2]. These symptoms, also referred to as computer vision syndrome, digital eyestrain, or occupational asthenopia, may be divided into two main groups[3–5]: internal or visual symptoms like blurred vision and diplopia and external or ocular symptoms like burning eyes and dry eyes.

Up until 2014, investigating CRVOS had the difficulty that there was no standardized, objective, reproducible and validated instrument for measuring these symptoms[6] and many studies used the questionnaire developed by Hayes *et al*.[1] which test-retest study is known[7] but whose psychometric properties, like validity or measurement precision, are unknown. In 2014, we presented the first scale for this purpose, the computer-vision symptom scale (CVSS17)[8] and in 2015 another Spanish group reported the computer vision syndrome questionnaire (CVS-Q)[9] which factor structure and/or levels of severity are unknown. The comparison of both questionnaires’ (CVSS17 and CVS-Q) psychometric properties shows that CVSS17 overcomes the main limitation of the CVS-Q -a suboptimal item-person targeting- and shows higher measurement precision.

The CVSS17, developed, validated and scored through Rasch analysis, was designed to provide a patient-reported measure of CRVOS among video display terminal (VDT) workers. The scale contains 17 items with scores ranging from 17 to 53 (a higher score indicates a greater level of symptoms) and is available in Spanish, English and Italian at http://www.cvss17.com. Any person can freely access the questionnaire and obtain a score right away. CVSS17 ensures construct validity and provides measures as a linear interval scale, measuring CRVOS without the main limitations of previously developed instruments [8]. Besides the construct validity, we studied [8, 10] different types of the CVSS17´s validity by assessing the association between the CVSS17 scores and other closely related questionnaires, like the Visual Discomfort Scale (VDS)[11] and the Ocular Surface Disease Index (OSDI)[12]. In addition, we tested the convergent validity[10] finding an association by which CVSS17 scores are higher as the amplitude of accommodation decrease and when the difference between the accommodative amplitude and the amplitude, measured by push-down method, increase.

To be useful, data provided by CVSS17 should be easy to interpret and actionable[13], i.e. scores guide diagnostic or therapeutic actions/decision making[14]. In respect to this topic, providing evidence-based thresholds that identify levels of symptoms is useful for the clinical management of patient [15] as it helps in the score interpretation[16].

Rasch analysis provides the measure and standard error corresponding to every possible raw score and can be used to determine how many statistically different levels exist across the score range[17], in questionnaires assessing symptoms these levels of performance represent the grades of symptoms severity measured. Therefore, this analysis provides proper cutoff values to transform CVSS17 scores into categorical.

The main factors of the CVSS17 were identified by conventional factor analysis in the initial validation of the scale[8]. Accordingly, CVSS17 shows a two-factor structure similar to other models proposed for CRVOS measured either by Hayes questionnaire[3] or by a checklist composed by some common CRVOS[18]; one factor is related to ocular dryness and the other is generally caused by refractive, accommodative or vergence anomalies[5]. However, to the best of our knowledge, the domain structure of these prior models has not been examined to verify whether their factors represent independent latent traits [19]. This is an important point in CROVS analysis because the strategies for diagnosis, management and/or research of these problems may differ depending on the relative importance of each main factor. In addition, grouping scale items within domains is essential because they can form subscales that allow for the assessment of CRVOS at more specific levels[20].

The present study was therefore designed to: 1) determine the number of different performance levels of CVSS17 using the method proposed by Wright [17] because is a sample-independent method more suitable for clinical samples, typically skewed to healthy or “sick” people, than the separation ratio provided by Rasch analysis which assumes that the test is targeted on the sample; 2) confirm through discriminant analysis if the main factors described in our prior work are capable on their own to classify subjects according to these performance levels; 3) to validate through Rasch analysis CVSS17 factors as independent latent traits able to measure the specific principal components of CRVOS.

## Methods

The study protocol adhered to the tenets of the Declaration of Helsinki and was approved by the Research Ethics Committee of the Hospital Clínico San Carlos (Madrid, Spain). We obtained electronic informed consent the participants before accessing the questionnaire’s webpage and before sending the responses.

All p-values provided are two-tailed. Significance was set at p<0.05.

### Participants

The following subjects were invited to complete the CVSS17 online over the period May 2012 to November 2013: the members of a trade union (*Unión General de Trabajadores*), the partners of a health and safety at work organization (*Grupo OTP- Prevención de Riesgos Laborales*), the workers of a private company (SIEMENS) and of a public entity (Spanish National Institute of Statistics, INE).

Subjects were 18 to 65 years old, spoke Spanish, and fulfilled the definition of a VDT worker established by the *Instituto Nacional de Seguridad e Higiene en el Trabajo* (INSHT, Spanish Institute of Health and Safety at Work)[21]. Further inclusion criteria were no ocular disease or medication that could affect their vision. When fewer than 12 items (two-thirds) were answered and/or person outfit was over 2.5 the corresponding questionnaires were excluded from the analysis[8, 20]. Subjects indicating they were over 39 years of age were considered presbyopes. Furthermore, we took into account neither the refractive status nor any accommodative or binocular problems in the participants’ recruitment.

822 subjects agreed to complete the CVSS17 and, after applying inclusion and exclusion criteria, 26 questionnaires with Outfit > 2.5 were excluded so 796 were finally analyzed (age: 43.9 ± 10 years; 58.04% female; 35.66% non-presbyopes).

### Rasch analysis

The Rasch model is an item response theory (IRT) model. The model transforms raw scores to preserve the distance between the locations of two persons regardless of the particular items administered. The main IRT concept is that a mathematical model is used to predict the probability of a person successfully replying to an item according to the person’s ability and item difficulty[22]. Since the selected items were polychotomous, for Rasch analysis we had to choose between the partial credit model (PCM, which considers a different rating scale for each item) and the Andrich rating scale model (RSM, which assumes equal category thresholds across items). We chose PCM for the reasons provided in our previous paper [8].

### Levels of performance

We used the PCM results provided by WINSTEPS software (ver. 3.92.1, Chicago, IL) to estimate the CVSS17 measures (in logits) and standard errors corresponding to every possible raw score. We used these data to compute the number of significantly different levels of performance according to the methods proposed by Wright[17].

### Confirmatory factor analysis

We used the IBM SPSS Statistics package version 22.0 (Statistical Package for Social Sciences) for factor analysis of data from those subjects who answered every item to confirm the factorial structure of the items in the scale.

In the exploratory factor analysis of the CVSS17, published in 2014[8], the structure of the factor model was unknown; rather, data helped us to reveal or to identify the simplified structure given by the items in CVSS17. In the factor analysis presented in this paper, on the other hand, the precise structure of the factor model is hypothesized or known and would be confirmed with this analysis[23].

Discriminant analysis (DA) is a statistical multivariate technique useful to classify observation into different groups. The objective of DA is to identify the minimum number of discriminant functions that will provide most of the discriminations among the groups[23]. Given a set of independent variables, functions discriminates between individuals and allocates each of them to a group defined by a dependent categorical variable. Therefore, the basic problem in discriminant analysis is to assign an unknown subject to one of two or more groups on the basis of a multivariate observation[24]. DA differs from group building techniques in that the groups must be known in advance. This is the appropriate method if the independent variables are metric and the dependent variable is non metric[23]. Moreover, it is possible to determine how successful the classification is.

Thus, we used DA to examine if the two factors of the CVSS17 identified in the exploratory factor analysis could accurately classify subjects according to the previously defined levels of performance. To this end, subjects were first grouped according to their level of symptom severity. Then, the DA was developed for the obtained factors (independent variables) to confirm if factors discriminates between different severity levels (dependent variable). A large proportion of subjects correctly assigned to each group was taken to indicate the high discrimination power of the CVSS17.

### Domain structure assessment

For PCM analysis of each of the two CVSS17 domains (main factors), we used WINSTEPS software to assess the following properties for each domain:

1. Item fit statistics. Both Infit and Outfit mean square fit statistics show the extent to which the items in the domain comply with Rasch model expectation[25].
2. Dimensionality. The scale is considered unidimensional when there is one latent variable of interest, and the level of this latent variable is the focus of measurement[22]. Two parameters derived from principal component analysis (PCA) of standardized residuals are used for this assessment: the amount of raw variance explained by the measure and the eigenvalue of the unexplained variance in the first contrast[25].
3. Person separation index (PSI) and levels of performance. Rasch-based PSI is a reliability indicator, analogous to Cronbach’s α of traditional test theory in both values and construction[26]_._ This index was obtained through WINSTEPS. Levels of performance were computed as described above.
4. Targeting. This was established as the difference between the average difficulty of the items and subjects’ mean level of symptoms [25].
5. Differential Item Functioning (DIF). We examined each main factors’ items to check there was no difference in the way subgroups (male–female; presbyopes–nonpresbyopes) answered each item (i.e., no DIF). We used the DIF analysis implemented in WINSTEPS based on two methods:
  - Mantel-Haenszel method to estimate (log-) odds DIF size and significance from cross-tabs of observations in the two groups.
  - Logit-difference (logistic regression) method to estimate the difference between Rasch item difficulties for the two groups, maintaining everything else constant[27].

The quality of the data obtained in the domain structure assessment stage (except levels of performance) was assessed according to the criteria of the guidelines proposed by Khadka et al.[25] for quality assessment of ophthalmologic questionnaires.

## Results

CVSS17 scores were mean 31.31, median 31.0, minimum 17.0, maximum 50.0 and their standard deviation was 7.65. The 95% confidence interval for the population mean was 30.78 to 31.84. PCM summary statistics are provided in S1 Appendix.

The two main factors described by CVSS17 factor analysis was named in accordance to our previous paper, ESF and ISF[8].

We used the responses of the 600 subjects who answered every item to analyze the differences in CVSS17, ESF and ISF scores according to gender and age group (non-presbyope women, non-presbyope men, presbyope women and presbyope men) by Kruskal-Wallis test followed by Dunn's multiple comparisons test, because K-S test indicated a normal distribution neither for CVSS17 scores nor for the main factors’ scores. Kruskal-Wallis test showed that there was a significant difference between the analyzed groups for CVSS17 (H: 37.01, p<0.001), ESF (H: 34.08, p<0.001) and ISF (H: 33.51, p<0.001). According to median values (Table 1) and Dunn’s test results (Table 2), presbyope women showed significant higher values for CVSS17, ESF and ISF scores. No more significant differences were found.

**Table 1:** Descriptive statistics by age and gender group for CVSS17, External Symptom Factor (ESF) and Internal Symptom Factor (ISF).

	Non-presbyope women (NPW)	Non-presbyope men (NPM)	Presbyope women (PW)	Presbyope men (PM)	Total
	**CVSS17**				
**n**	135	64	219	182	600
**Median**	29	26.5	34	28	30
**Intercuartile range**	24 to 37	21,25 to 33,5	27 to 39	23 to 34,25	25 to 37
	**External Symptom Factor (ESF)**				
**n**	135	64	219	182	600
**Median**	19	17	21	18	11
**Intercuartile range**	15 to 34	14 to 22	17 to 26	15 to 23	16 to 24
	**Internal Symptom Factor (ISF)**				
**n**	135	64	219	182	600
**Mean**	10.93	9.66	11.93	10.6	11.06
**Intercuartile range**	8 to 13	8 to 11	9 to 14	8 to 13	8 to 13

**Table 2:** p-values obtained in Dunn’s post hoc tests for pairwise comparisons results.

	CVSS17			ESF			ISF		
	NPM	PW	PM	NPM	PW	PM	NPM	PW	PM
**NPW**	0.052	<0.001*	1,000	0.110	0.020*	1,000	0.052	0.018*	1,000
**NPM**		<0.001*	0.439		<0.001*	0.889		<0.001*	0.216
**PW**	<0.001*		0.014*	<0.001*		<0.001*	<0.001*		<0.001*

NPW, NPM, PW and PM are defined in Table 1a heading

* indicates a p-value lower than 0.05

### Levels of performance

Rasch analysis revealed 5.8 different levels of performance and a level reliability of 0.97 (see S2 Appendix for details). CVSS17 performance levels (symptom severity grades) and subject distributions across these levels are detailed in Table 3.

**Table 3:** CVSS17 levels of performance (symptoms severity).

	CVSS17 levels of performance					
	Level 1	Level 2	Level 3	Level 4	Level 5	Level 6
**Score range**	[17,23)	[23,29)	[29,36)	[36,43)	[43,49)	[49,53]
**n (%)**	119 (14.95)	191 (24.00)	245 (30.78)	180 (22.61)	54 (6.78)	7 (0.88)

Score intervals defining each level are described in the first row and the number of subjects (and percentage) classified among each level in the bottom row.

**Fig 1:**
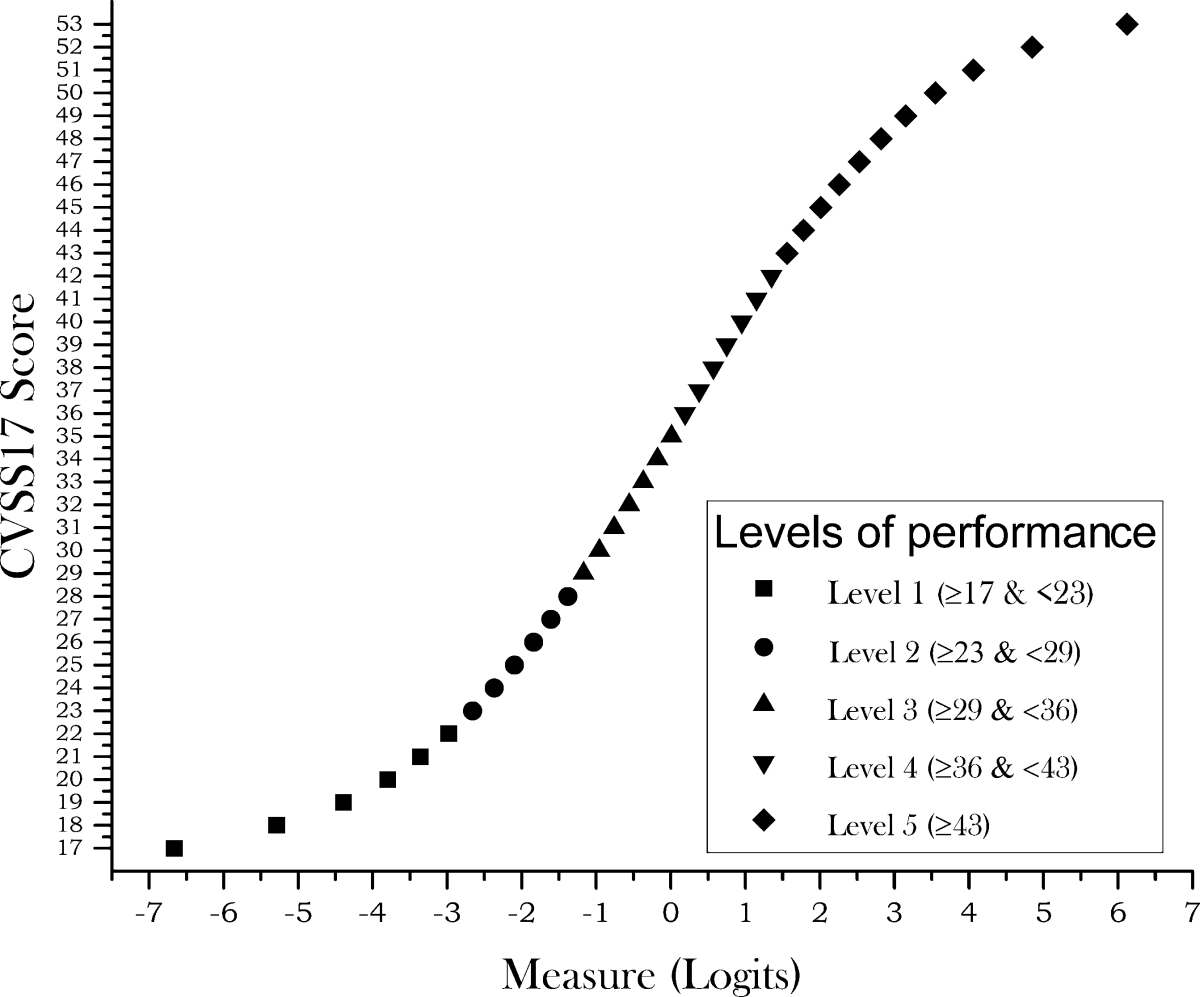
Plot of the estimated measure for any CVSS17 raw score. Plot of the estimated measure (x-axis) for any raw CVSS17 score (y-axis). Different symbols represent distinct levels of performance as indicated in the figure inset.

As only seven subjects were allocated to the top level (level 6), levels 5 and 6 were collapsed so five levels were finally defined (Fig 1).

### Confirmatory factor analysis

For this analysis, we selected questionnaires without missing responses corresponding to 600 subjects (age: 44.4 ± 10; 59.0% female; 33.2% non-presbyopes). CVSS17 scores for these subjects were mean 30.87, median 30.0, minimum 17.0, and maximum 50.0; standard deviation was 7.82.

**Fig 2:**
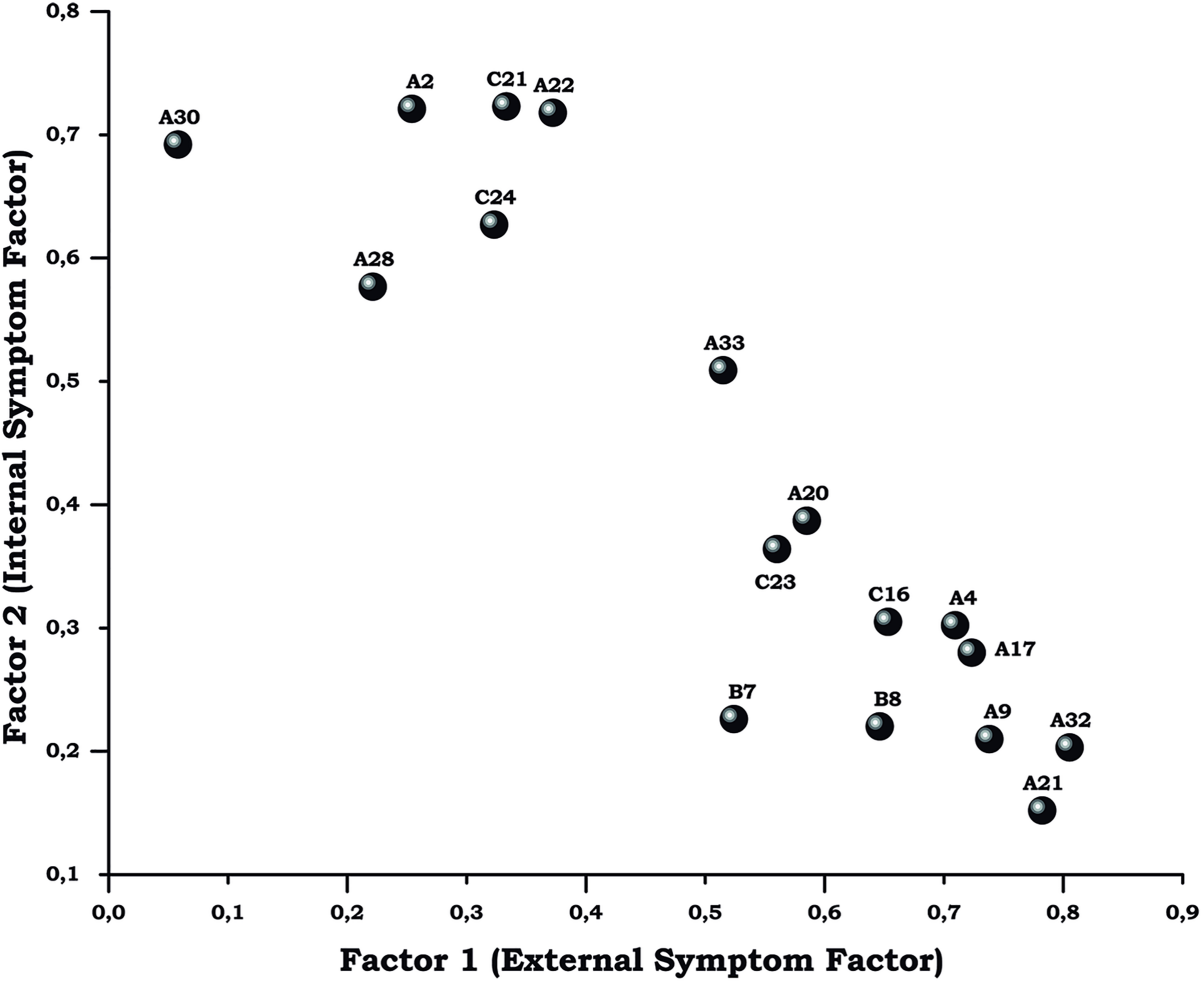
Factor loadings for CVSS17 principal components. Plot of the factor is loading for Factor 1 (external symptom factor, horizontal axis) against Factor 2 (internal symptom factor, vertical axis) for each of the CVSS17 items. Item descriptors are shown in Table 4.

First, as the Bartlett's sphericity test showed significance, the Kaiser-Meyer-Olkin (KMO) index was used to verify if our data were suitable for factor analysis. As KMO was 0.94, factor analysis was performed and the number of principal components determined by selecting factors with eigenvalues over one. A two-factor structure (rotated component matrix is shown in Table 4 and a graph showing the factor loading for each one of the principal components in Fig 2) accounted for 53.87% of the total variability.

**Table 4:** Rotated components matrix.

Item descriptor	Component	
	1	2
**A32. Did your eyes sting?**	0.80	0.21
**A21. Did your eyes burn?**	0.78	0.15
**A9. Did your eyes hurt?**	0.74	0.22
**A17. After working on the computer for a while did your eyes become heavy?**	0.71	0.30
**A4. Did your eyes become tired?**	0.68	0.34
**C16. At the end of my working day, my eyes feel heavy**	0.67	0.21
**B8. Eye redness**	0.63	0.34
**A20. Did you have to blink more than usual?**	0.59	0.38
**C23. While I’m working, I have to close my eyes to relieve eye dryness**	0.56	0.36
**B7. Watery Eyes**	0.53	0.24
**A33. After working on the computer for a while did lights bother you?**	0.48	0.54
*C21*. *After working at the computer*, *I have to strain to see well*	0.37	0.72
*A2*. *Did the letters on the screen become blurry*?	0.33	0.73
*A22*. *Did you have to strain to see well*?	0.30	0.67
*A30*. *Did the letters appear double*?	0.25	0.73
*C24*. *After some time at the computer*, *lights bother me*	0.24	0.57
*A28*. *Did you feel like you were crossing your eyes*?	0.08	0.68

Extraction method was principal-component factors and rotation method was Varimax with kaiser normalization. The two columns on the right shows each component's loading and items are displayed on the left column, ordered by component 1 loading. Items with factor 1's loading are highlighted in bold and those with factor 2's loading over 0.5 are in cursive

Once we had identified the principal components, a univariate descriptive analysis was conducted by calculating the means and standard deviations of the scale's main factors separately for each performance group (Table 5).

**Table 5:** Univariate descriptive analysis results.

CVSS17 Level	Recoded factor score	Mean	S.D.	n
**1**	**Factor 1**	-1.22	0.38	101
	**Factor 2**	-0.67	0.25	101
**2**	**Factor 1**	-0.49	0.51	157
	**Factor 2**	-0.48	0.59	157
**3**	**Factor 1**	0.21	0.74	167
	**Factor 2**	0.01	0.88	167
**4**	**Factor 1**	0.87	0.78	130
	**Factor 2**	0.55	1.05	130
**5**	**Factor 1**	1.17	0.65	45
	**Factor 2**	1.55	0.90	45
**Total**	**Factor 1**	0.00	1.00	600
	**Factor 2**	0.00	1.00	600

Mean and standard deviation are shown for each recoded factor score among every performance group (left column), Number of valid responses are shown in the right column.

**Fig 3:**
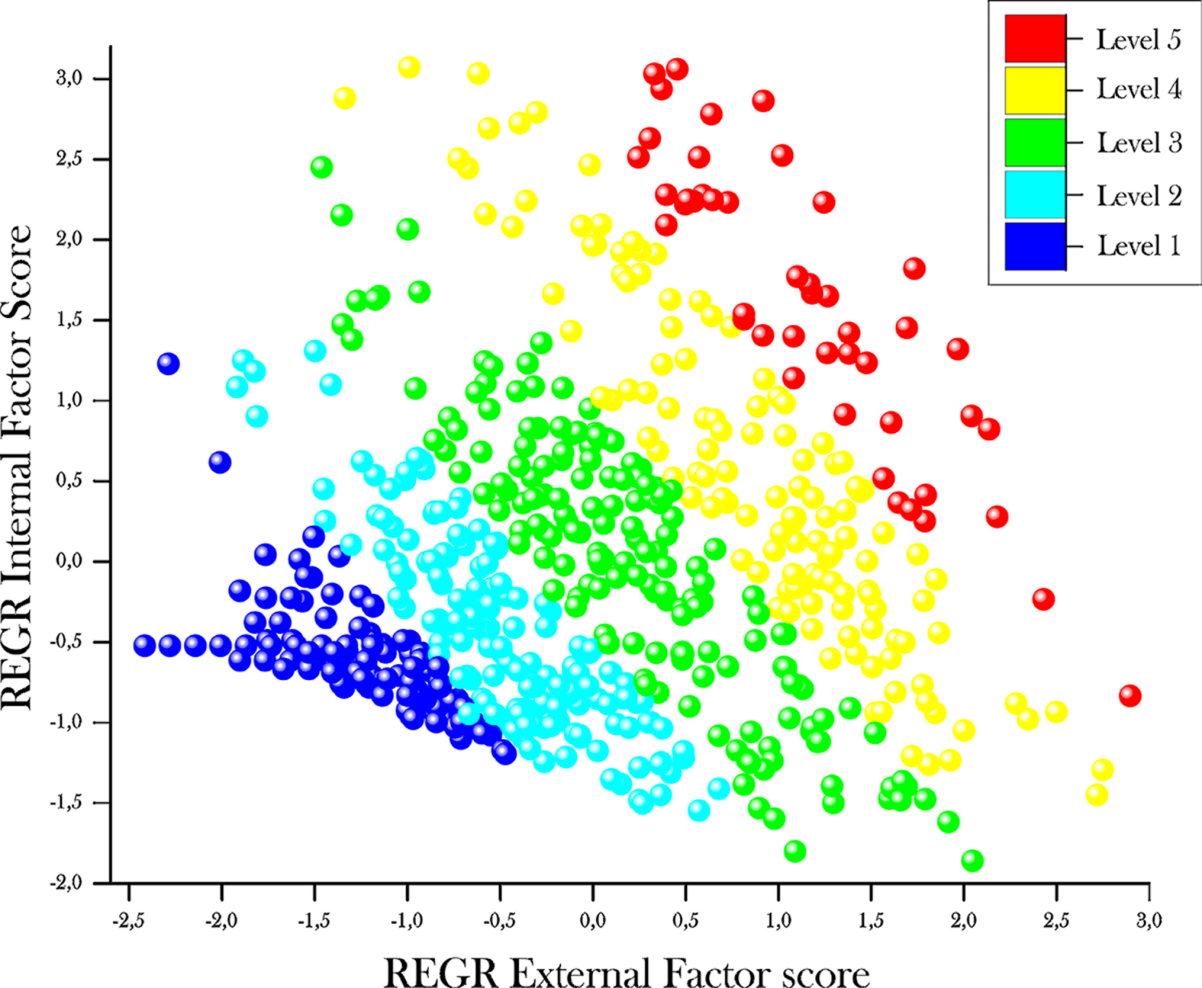
Discriminant analysis scatter plot of the two factors model. Discriminant analysis scatter plot of the two factors model. Recoded external factor scores (x-axis) are plotted against recoded internal factor scores (y-axis). Different grey intensities represent distinct subject levels of performance as indicated in the figure inset.

As shown in Table 5, mean factor values differed across performance levels and their variability is less than 1 in most cases, indicating that the scale's main factors could be good at discriminating between groups. This result is confirmed with the DA concluding that the mean of at least one pair of groups are significantly different (p<0.05 for both factors). Fig 3 shows the factor loadings for each subject according to subject level of performance.

In addition, the discriminant functions obtained from the main factors were able to correctly classify 98.3% of the cases examined.

### Domain structure assessment

According to previously used nomenclature [4, 18], hereafter items with a Factor 1 loading (see Table 4) over 0.5 are referred to as an ESF and items with a Factor 2 loading over 0.5 as an ISF. Rasch analysis results are provided separately for ESF and ISF:

#### Rasch analysis results for ESF

**Fig 4:**
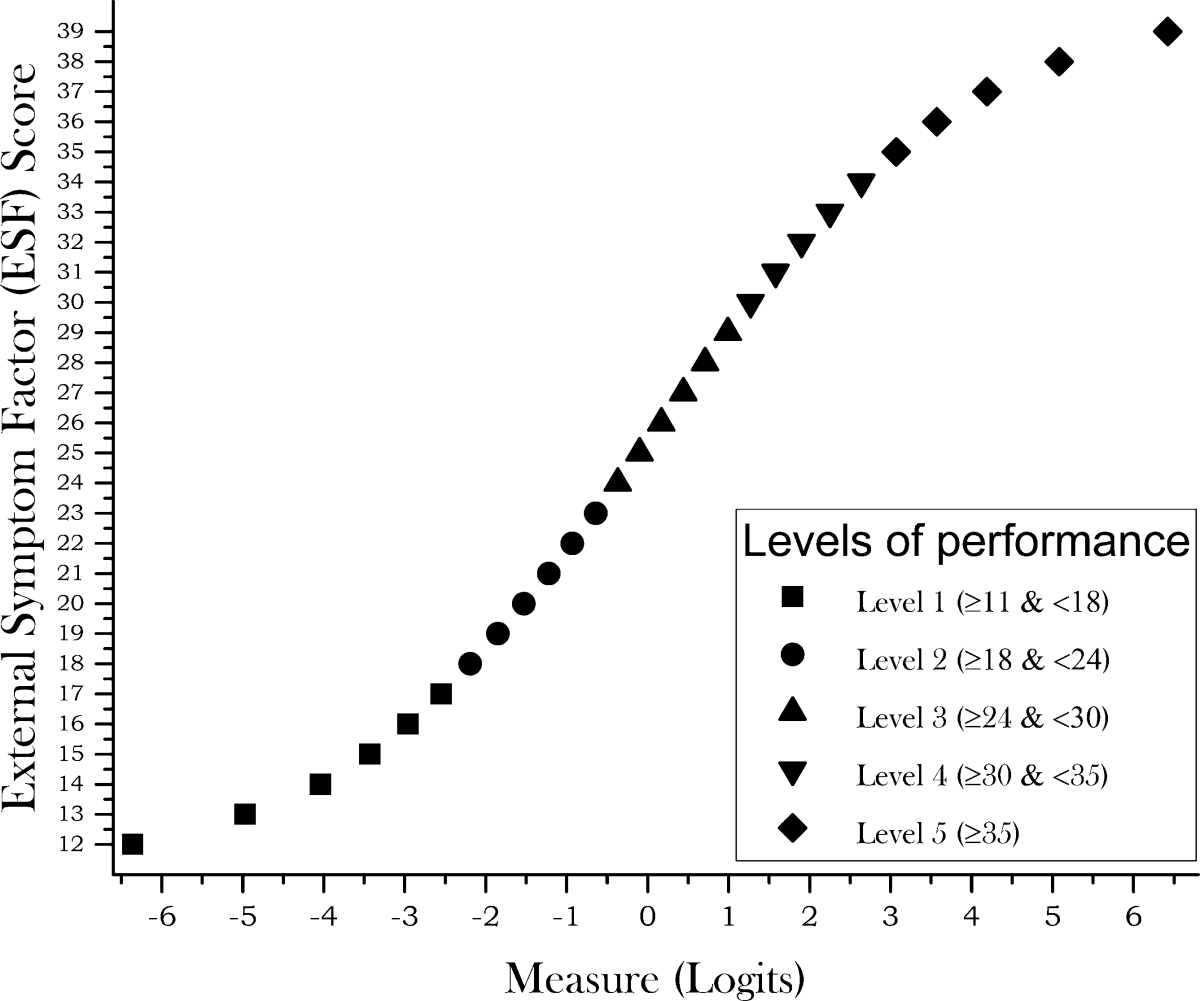
Plot of the estimated measure for any ESF raw score. Plot of the estimated measure (x-axis) for any ESF raw score (y-axis). Different symbols represent distinct levels of performance as indicated in the figure inset.

Infit and outfit mean squares were 0.99 and 1.00 respectively for persons, and 1.00 and 1.01 respectively for items. The eigenvalue of the unexplained variance in the first PCA contrast was 1.71 and raw variance explained by measures was 53%. PSI was 2.61 and there were 4.7 statistically different levels of performance (Fig 4). The difference between the average difficulty of the items and subjects was -0.41 logits. DIF for gender and age group was under 0.5 logits for all items. Table 6 shows our quality assessment of these results.

**Table 6:** Quality evaluation of data obtained in the External Symptom Factor (ESF) domain assessment.

Property	Evaluated variable	Result	Quality grade^Ɨ^ (khadka *et al*.)[25]
**Item fit Statistics**	**Number of Items with Infit outside (0.7,1.3)**	0	A
	**Number of Items with Outfit outside (0.7,1.3)**	0	A
**Dimensionality**	**Raw variance explained by measure**	53.0%	B
	**Eigenvalue of the first contrast**	1.71	B
**Measurement precision**	**Person separation index**	2.61	A
	**Number of statistically different levels (Reliability)**	4,7 (0,96)	A^Ɨ Ɨ^
**Targeting**	**Items dificulty - Subjects ability**	-0.41	A
**DIF**	**Number of items with DIF by gender > 0,5**	0	A
	**Number of items with DIF by age group > 0,5**	0	A

Ɨ A: High; B: Medium; C: Low.

Ɨ Ɨ Criteria was proposed for empirical reliability derived from Rasch-based person separation index, but in the present study this variable assessment was based on the sample-independent reliability provided by methods described by Wright[17]

### Rasch analysis results for ISF

**Fig 5:**
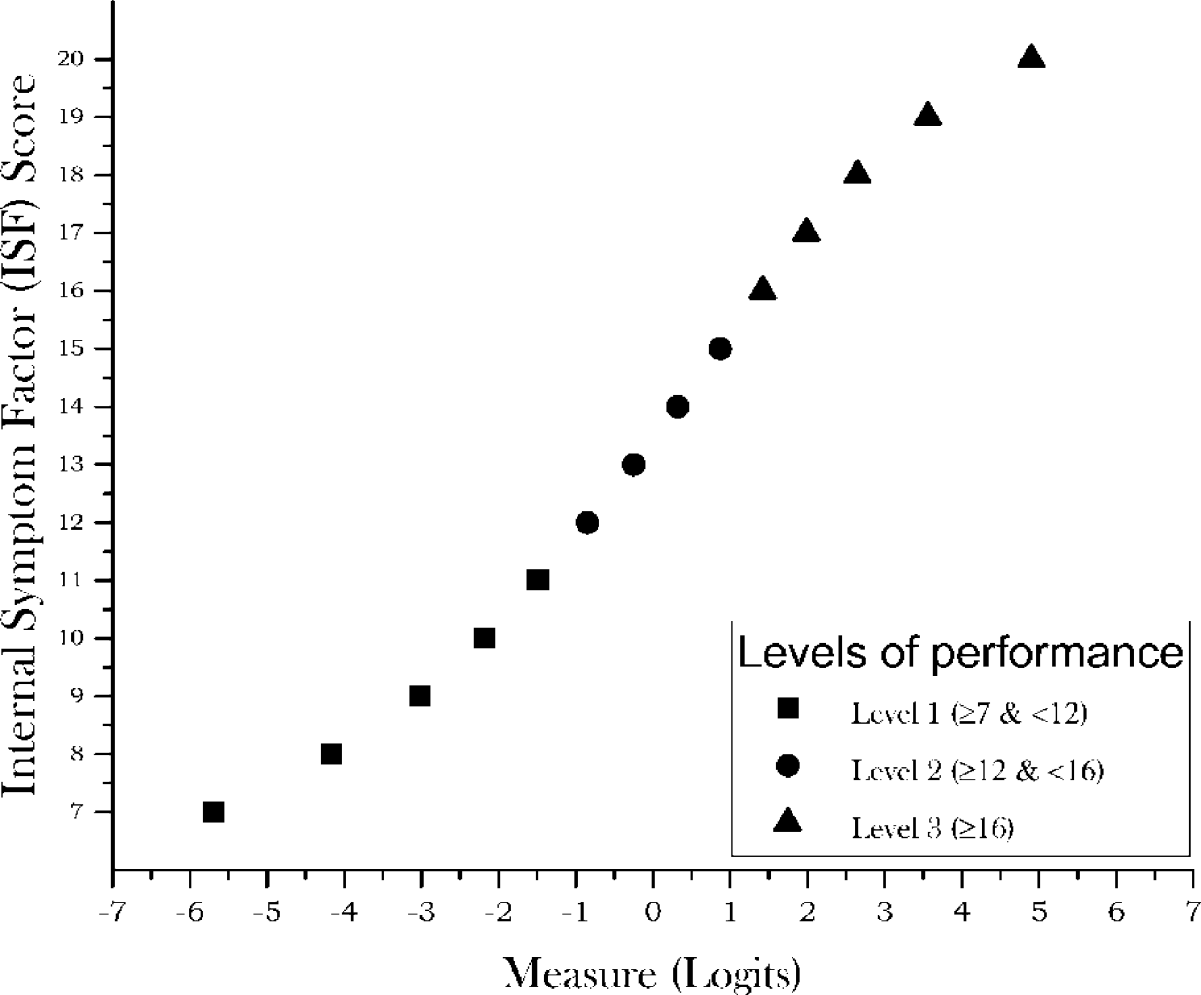
Plot of the estimated measure for any ISF raw score. Plot of the estimated measure (x-axis) for any ISF raw score (y-axis). Different symbols represent distinct levels of performance as indicated in the figure inset.

Infit and outfit mean squares were 0.99 and 0.99, respectively, for persons and 0.99 and 0.99 respectively for items. The eigenvalue of the unexplained variance in the first PCA contrast was 1.78 and raw variance explained by measures was 56.1%. PSI was 1.63 and there were 3.3 statistically different levels of performance (Fig 5). The difference between the average difficulty of the items and subjects was -1.27 logits. DIF for gender and age group was under 0.50 logits for all items. Table 7 shows our quality assessment of these results.

**Table 7:** Quality evaluation of data obtained in the Internal Symptom Factor (ISF) domain assessment.

Property	Evaluated variable	Result	Quality grade^Ɨ^ (khadka *et al*.)[25]
**Item fit Statistics**	**Number of Items with Infit outside (0.7,1.3)**	0	A
	**Number of Items with Outfit outside (0.7,1.3)**	2	B
**Dimensionality**	**Raw variance explained by measure**	56.1%	B
	**Eigenvalue of the first contrast**	1.78	B
**Measurement precision**	**Person separation index**	1.63	C
	**Number of statistically different levels (Reliability)**	3,3 (0,92)	A^ƗƗ^
**Targeting**	**Items dificulty—Subjects ability**	-1.27	B
**DIF**	**Number of items with DIF by gender > 0,5**	0	A
	**Number of items with DIF by age group > 0,5**	0	A

Ɨ A: High; B: Medium; C: Low.

ƗƗ Criteria was proposed for empirical reliability derived from Rasch-based person separation index, but in the present study this variable assessment was based on the sample-independent reliability provided by methods described by Wright[17]

## Discussion

This study identified five significantly different levels of symptoms among CVSS17 scores and confirmed the two-factor structure (ESF and ISF) of this scale. By Rasch analysis, it was also observed that ESF and ISF perform well as separate scales.

Because CVSS17 is a PRO instrument without DIF for gender and/or age group, we could directly compare the CRVOS among these subgroups, so our results describing a higher level of CRVOS in women and in presbyopes, despite previously reported[4, 5], are worthy of note and future studies should consider these differences in their analysis and should go depth in the research on the reasons that provokes a higher level of CRVOS in presbyope women.

Our analysis detected 5.8 levels of performance or symptoms across the scale's score range corresponding to a sample-independent reliability of 0.97. For easy interpretation of CVSS17 scores, we propose five levels of symptom severity whereby categories four and five indicate a higher level of subject symptoms than scale difficulty. This means that VDT workers assigned to these levels warrant priority attention. To our knowledge, no similar CRVOS scale shows such a good grading power, which was comparable to that calculated using the same methods for the Chinese Impact of Vision Impairment (IVI) questionnaire[28].

By discriminant analysis, we confirmed that the CVSS17’s main factors (ESF and ISF) can correctly classify subjects’ CRVOS according to their level of symptom severity. A reduced blink rate associated with computer use, a high cognitive load and low contrast reading conditions lead to ESF symptoms[29–31], while refractive errors, glare, accommodation system stress[29] and increased convergence[31] may cause ISF symptoms. As mentioned previously [8], other authors [3, 18] have proposed similar bifactorial models with differences in the factors assigned to eyestrain and photophobia. To explain these differences, it should be noted that the items included in a questionnaire determine factor composition. Sheedy *et al*[3]. selected nine symptoms measured using visual analogue scales (VAS) on a study sample of twenty students and University staff members, while Portello et al[3]. measured symptoms among VDT workers with the questionnaire developed by Hayes *et al*.[1] based on clinical findings and questionnaires used in the care of computer-using patients. The items included in CVSS17 were selected through Rasch analysis conducted on a population of VDT workers. Item A33 related to light-induced ocular discomfort showed a similar ESF and ISF factor loading. Thus, it could be that the pathophysiological mechanism that produces ocular discomfort associated with bright light may have components of both ESF and ISF.

ESF items are able to assess dry-eye symptoms related to computer use among VDT workers. In fact, when comparing their measurement properties against those recently described for the Ocular Comfort Index (OCI), Ocular Surface Disease Index (OSDI) and McMonnies Questionnaire [19] it emerged that ESF had benefits including better-fit statistics, a lower eigenvalue of the first PCA contrast, higher person separation and better items-person targeting. Accordingly, ESF could be the best option to assess dry eye symptoms in VDT workers. However, clinical research is still needed to confirm its separate performance by assessing other properties like repeatability and convergent validity. In addition, to help clinicians managing CRVOS, more research is needed to precise the relationship between clinical findings and the levels of severity and/or the subscales described in the present paper besides the associations described by us in a previous work between CVSS17 scores and some clinical measures that are summarized in Table 8.

**Table 8:** Coefficients of correlation between CVSS17 and some clinical measures, previously reported [10].

	CVSS17	ESF	ISF
**VDS**	0.66**^†^	0.58**^††^	0.72**^††^
**OSDI**	0.65**^†^	0.63**^††^	0.55**^††^
**AA**	0.34*^†^	n.s.	0.34*^†^
**DIFAA**	0.37*^†^	n.s.	-0.42**^†^
**M**_**R**_	n.s	n.s.	0.23**^††^
**J0**_**R**_	0.15*^††^	n.s.	0.15*^††^

**VDS =** Visual Discomfort Scale. **OSDI =** Ocular Surface Disease Index. **AA =** Amplitude of accommodation measured by push-down method. **DIFAA =** AA–Mean amplitude of accommodation predicted by Hofstetter formula. **M**_**R**_ **=** M component of the ocular refraction, measured by retinoscopy. **J0**_**R**_ = J0 component of the ocular refraction measured by retinoscopy. n.s. = non-significant

* = p<0.05

** = p < .01

† = Pearson's r

†† = Spearman's rho

The results displayed on Table 8 provide some evidence about the CVSS17’s concurrent validity but we still need to determine the way in which CRVOS vary as some clinical measures, like uncorrected refractive errors or tear film osmolarity, change. To do so, researchers need to compare valid and reliable clinical data against a valid and reliable model of CRVOS, like the one provided by the CVSS17.

According to the PSI, ISF measurement precision was below the acceptable limit given our participant distribution was skewed towards the less symptomatic part of the scale. However, by assessing reliability using the Wright method, we confirmed that ISF could distinguish three strata so at least it can discriminate between high and low performers. We therefore propose this subscale could be useful to assess internal symptoms among VDT workers. Notwithstanding, its reliability and persons-item targeting could be improved by adding more low-difficulty items.

Although more work is needed to compare changes in CVSS17 scores indicating clinically meaningful variations[32], our data suggest that a change in CVSS17 performance level may be perceived by a subject. The results showed in the present paper indicate that, besides the CRVOS level provided by the CVSS17, ESF or ISF are valid measures when defining the optimal assessment strategy and/or treatment for any patient. Table 9 depicts an example of subscales-guided clinical decisions taken from real CVSS17 scores, where Person 1 and Person 2 score is the same, 37, but just looking at their subscales’ scores the clinician would focus on dry eye when dealing with Person 1 but would consider ocular refraction, accommodation and/or vergence anomalies when caring for Person 2.

**Table 9:** Example for subscales’ scores interpretation.

	CVSS17 Score	CVSS17Level	ESF Score	ESFLevel	ISF Score	ISFLevel
*Person 1*	37	4	31	4	10	1
*Person 2*	37	4	20	2	18	3

We have to point out that we assessed neither the participants’ refractive status nor their accommodative or binocular anomalies so an under/overrepresentation of these anomalies in our sample could lead to an under/overestimation of values like the CVSS17 population mean, the median values or the number of subjects in each level of performance but it has no effect in the main findings of the study like the factor structure of the CVSS17 or in the levels of performance cut-off scores.

CVSS17 was originally developed and validated in Spanish. Since its development, other research groups have started its cross-cultural adaptation to English, Italian and Portuguese. For a better understanding of the CVSS17’s items, we provide the English printable versions of CVSS17, ESF and ISF along with the original Spanish versions and their scoring charts (S3, S4, S5, S6, S7, S8, S9, S10 and S11 Appendix). To help clinicians and researchers willing to use the CVSS17, we include a spreadsheet in English (S12 Appendix) and in Spanish (S13 Appendix) that automatically provide the CVSS17, ESF and ISF score after entering manually the answers to the CVSS17 questionnaire. In addition, data used for this research is provided in S1 Dataset.

In conclusion, CVSS17 is a highly reliable PRO (patient reported outcomes) tool to assess CRVOS in VDT workers, with scores defining five different levels of performance. In addition, two main factors (ESF and ISF) were identified through factor analysis. These main factors or subscales were confirmed by discriminant analysis and are consistent with our previous findings. Accordingly, clinicians and/or researchers could separately use the ESF and ISF subscales to assess the specific components of CRVOS.

## Supporting information

S1 Appendix Summary statistics for Rasch analysis. (PDF)

S2 Appendix CVSS17 levels of severity calculation. (PDF)

S3 Appendix Printable English version of the CVSS17. (PDF)

S4 Appendix Printable Spanish version of the CVSS17. (PDF)

S5 Appendix Scoring chart of the CVSS17. (PDF)

S6 Appendix Printable English version of the CVSS17-ESF. (PDF)

S7 Appendix Printable Spanish version of the CVSS17-ESF. (PDF)

S8 Appendix Scoring chart of the CVSS17-ESF. (PDF)

S9 Appendix Printable English version of the CVSS17-ISF. (PDF)

S10 Appendix Printable Spanish version of the CVSS17-ISF. (PDF)

S11 Appendix Scoring chart of the CVSS17-ISF. (PDF)

S12 Appendix Spreadsheet for entering and scoring CVSS17 responses (English). (XLSX)

S13 Appendix Spreadsheet for entering and scoring CVSS17 responses (Spanish). (XLSX)

S1 Dataset Spreadsheet showing each subject’s responses to each CVSS17 item. (XLSX)
