# Supplementary material for: Five levels of performance and two subscales identified in the computer-vision symptom scale (CVSS17) by Rasch, factor, and discriminant analysis
Source: PLoS One. 2018 Aug 28;13(8):e0202173. doi: 10.1371/journal.pone.0202173 (PMC6112632; doi:10.1371/journal.pone.0202173)
Supplement: S1 Appendix — (PDF) [file pone.0202173.s001.pdf]

SUMMARY OF 814 MEASURED (NON-EXTREME) PERSONS

	Raw Score	Count	Measure	Model Error	Infit		Outfit	
					MNSQ	ZSTD	MNSQ	ZSTD
**Mean**	30.75	16.7	-0.85	0.51	0.99	-0.1	0.99	0.0
**P.SD**	7.38	0.7	1.70	0.11	0.43	1.2	0.52	1.1
**S.SD**	7.38	0.7	1.70	0.11	0.43	1.2	0.52	1.1
**Max.**	50.0	17.0	3.54	1.10	3.06	3.9	3.79	3.8
**Min.**	17.0	13.0	-5.28	0.43	0.23	-3.2	0.13	-3.1

**Real RMSE**	0.56	**Adj. SD**	1.62	**Separation**	2.90	**Person Reliability**	0.89
**Model RMSE**	0.52	**Adj. SD**	1.63	**Separation**	3.15	**Person Reliability**	0.91

Standard Error of subjects mean= 0.06

Table 1a. **Summary statistics for Rasch Analysis (Persons). Raw Score** is the number of correct responses excluding extreme scores, **Count** is the number of responses made, **Measure** is the estimated measure (for persons), **Max** is its maximum value, **Min** is its minimum value **Real/Model: Real** is computed on the basis that misfit in the data is due to departures in the data from model specifications, **Model** is computed on the basis that the data fit the model, and that all misfit in the data is merely a reflection of the stochastic nature of the model, **SE** is the standard error of the estimate, **Infit** is an information-weighted fit statistic, which is more sensitive to unexpected behavior affecting responses to items near the person's measure level, **MNSQ** is the mean-square infit statistic with expectation 1, **ZSTD** is the infit mean-square fit statistic t standardized to approximate a theoretical mean 0 and variance 1 distribution, **Outfit** is an outlier-sensitive fit statistic, more sensitive to unexpected behavior by persons on items far from the person's measure level, **Mean** is the average value of the statistic, **P.SD** is its standard deviation assuming that this sample of the statistic is the entire population. It is not, the corrected sample S.D. (**S.SD**) = (P.SD / √ (Count of statistic) / (Count of statistic - 1)) , **Adj.SD** is the standard deviation of the estimates after subtracting from their observed variance the error variance attributable to their standard errors of measurement, **Separation** coefficient is the ratio of the PERSON (or ITEM) TRUE P.SD, the "true" standard deviation, to RMSE, the error standard deviation and **Person (or Items) Reliability** is a separation reliability (separation index). The PERSON (or ITEM) reliability is equivalent to KR-20, Cronbach Alpha, and the Generalizability Coefficient

SUMMARY OF 17 MEASURED ITEMS

	Raw Score	Count	Measure	Model Error	Infit		Outfit	
					MNSQ	ZSTD	MNSQ	ZSTD
**Mean**	665.6	786.8	0.00	0.07	1.00	0.0	0.99	-0.3
**P.SD**	291.1	24.1	0.89	0.01	0.08	1.7	0.10	1.4
**S.SD**	300.1	24.8	0.92	0.01	0.09	1.7	0.10	1.4
**Max.**	1220.0	802.0	1.86	0.12	1.17	2.7	1.18	2.5
**Min.**	106.0	718.0	-1.63	0.06	0.88	-2.5	0.85	-2.7

**Real RMSE**	0.08	**Adj. SD**	0.88	**Separation**	11.76	**Items Reliability**	0.99
**Model RMSE**	0.07	**Adj. SD**	0.88	**Separation**	11.98	**Items Reliability**	0.99

Standard Error of CVSS17 item mean= 0.22

Table 1b. **Summary statistics for Rasch Analysis (Items)**. **Measure** is the estimated measure (for items)
