# Supplementary material for: Five levels of performance and two subscales identified in the computer-vision symptom scale (CVSS17) by Rasch, factor, and discriminant analysis
Source: PLoS One. 2018 Aug 28;13(8):e0202173. doi: 10.1371/journal.pone.0202173 (PMC6112632; doi:10.1371/journal.pone.0202173)
Supplement: S2 Appendix — (PDF) [file pone.0202173.s002.pdf]

Raw Score	Measure in logits	Standard Error	Minimum measure for next level (must be less than Logit Measure to start the next level)	Level
17	-6,66	1,89	-6,66	1
18	-5,29	1,11	-2,28	1
19	-4,39	0,83	-2,53	1
20	-3,8	0,71	-2,62	1
21	-3,36	0,64	-2,67	1
22	-2,98	0,59	-2,70	1
23	-2,66	0,55	-2,72	2
24	-2,37	0,53	-1,13	2
25	-2,1	0,51	-1,16	2
26	-1,84	0,49	-1,19	2
27	-1,61	0,48	-1,20	2
28	-1,38	0,47	-1,21	2
29	-1,17	0,46	-1,23	3
30	-0,96	0,45	0,12	3
31	-0,76	0,44	0,10	3
32	-0,56	0,44	0,10	3
33	-0,37	0,43	0,09	3
34	-0,18	0,43	0,09	3
35	0,01	0,43	0,09	3
36	0,19	0,43	0,09	4
37	0,38	0,43	1,41	4
38	0,57	0,43	1,41	4
39	0,75	0,44	1,42	4
40	0,95	0,44	1,42	4
41	1,15	0,45	1,43	4
42	1,35	0,45	1,43	4
43	1,56	0,46	1,45	5
44	1,78	0,47	2,88	5
45	2,01	0,49	2,90	5
46	2,26	0,51	2,93	5
47	2,53	0,53	2,96	5
48	2,82	0,56	3,01	5
49	3,15	0,6	3,07	6
50	3,55	0,66	4,93	6
51	4,06	0,78	5,12	6
52	4,85	1,05	5,57	6
53	6,12	1,85	7,04	6

S2 Appendix. CVSS17 levels of severity calculation. Once the standard errors have been computed, they are used to compute how many statistically different levels of performance can be identified. The table above is based on Winsteps Table 20. To do this start at one end of the raw score range and work to toward the other. Advance each time by twice the joint standard error (= square-root of sum of squared standard errors) of the current starting and ending measures until there is not room for another level. In the Table, there are 5.8 statistically distinct levels of performance. This corresponds to a sample-independent reliability of at least $(5,8)^2/[1+(5.8)^2]$ = 0.97. The empirical reliability of this test was 0.89, a value which under-reports the test's measurement effectiveness.
