# Supplementary material for: Five levels of performance and two subscales identified in the computer-vision symptom scale (CVSS17) by Rasch, factor, and discriminant analysis
Source: PLoS One. 2018 Aug 28;13(8):e0202173. doi: 10.1371/journal.pone.0202173 (PMC6112632; doi:10.1371/journal.pone.0202173)
Supplement: S6 Appendix — (PDF) [file pone.0202173.s006.pdf]

FACULTAD DE ÓPTICA Y OPTOMETRÍA
UNIVERSIDAD COMPLUTENSE DE MADRID
C/ ARCOS DE JALÓN 118 - 28037 MADRID - ESPAÑA

CVSS17

Name, Surname:____

Age:___ **Date:**_________

FOLLOWING QUESTIONS ASK ABOUT HOW YOU FELT DURING YOUR LAST FOUR WORKING WEEKS:

If you normally wear glasses or contact lenses during most of your working hours, answer as if you were wearing them.
Please, circle your preferred choice in each question.

A4. Have you felt your eyes tired during or after working with your computer?

1. Never 2. Almost never 3. Seldom 4. Ocassionally

5. Frequently 6. Almost always 7. Always

A9. Did your eyes hurt when working with you computer?

4. Always 3. Frequently 2. Rarely 1. Never

A17. Have you noticed your eyes heavy after some time working with your computer?

4. Always 3. Frequently 2. Rarely 1. Never

A20. Did you have to blink a lot while using the computer at work?

1. Never 2. Rarely 3. Frequently 4. Always

A21. Did you experience burning eyes?

4. Always 3. Frequently 2. Rarely 1. Never

A32. How frequently have you noticed stinging in your eyes while working on the computer?

1. Never 2. Rarely 3. Frequently 4. Always

A33. Have you noticed that the lights bother you after some time on the computer?

1. Never 2. Almost never 3. A few times

4. Several times 5. Often 6. Very Often

Now, regarding your experience during the last four working weeks, please indicate to what extent you've felt the following troubles:

If you normally wear glasses or contact lenses during most of your working hours, answer as if you were wearing them.

	None (1)	Very little(2)	Litte (3)	A moderate amount (4)	Much (5)	Very much (6)
**B7. Watery eyes**	○	○	○	○	○	○
**B8. Eye redness**	○	○	○	○	○	○

To finish, please indicate to what extent you consider true or false each one of the following statements. If you normally wear glasses or contact lenses during most of your working hours, answer as if you were wearing them.

C16. At the end of my working day, I feel heavy eyes

1. Quite false 2. Completely false

3. Quite true 4. Completely true

C23. While I'm working, I have to close my eyes to relieve eye dryness

4. Completely true
3. Quite true
1. Quite false
2. Completely false
