# Supplementary material for: Five levels of performance and two subscales identified in the computer-vision symptom scale (CVSS17) by Rasch, factor, and discriminant analysis
Source: PLoS One. 2018 Aug 28;13(8):e0202173. doi: 10.1371/journal.pone.0202173 (PMC6112632; doi:10.1371/journal.pone.0202173)
Supplement: S8 Appendix — (PDF) [file pone.0202173.s008.pdf]

	Response Option						
**Ítem Id.**	***1***	***2***	***3***	***4***	***5***	***6***	***7***
A4	1	1	2	2	3	3	3
A9	1	2	3	4			
A17	1	2	3	4			
A20	1	2	3	4			
A21	1	2	3	3			
A32	1	2	3	4			
A33	1	2	2	3	3	3	
B7	1	1	2	2	2	2	
B8	1	1	2	2	3	3	
C16	1	1	2	3			
C23	1	1	2	3			

CVSS17 Score = [(Sum of scores) x 11] / (number of valid responses)
