# Supplementary material for: Five levels of performance and two subscales identified in the computer-vision symptom scale (CVSS17) by Rasch, factor, and discriminant analysis
Source: PLoS One. 2018 Aug 28;13(8):e0202173. doi: 10.1371/journal.pone.0202173 (PMC6112632; doi:10.1371/journal.pone.0202173)
Supplement: S9 Appendix — (PDF) [file pone.0202173.s009.pdf]

FACULTAD DE ÓPTICA Y OPTOMETRÍA
UNIVERSIDAD COMPLUTENSE DE MADRID
C/ ARCOS DE JALÓN 118 - 28037 MADRID - ESPAÑA

CVSS17

Name, Surname:____

Age:___ **Date:**_________

FOLLOWING QUESTIONS ASK ABOUT HOW YOU FELT DURING YOUR LAST FOUR WORKING WEEKS:

If you normally wear glasses or contact lenses during most of your working hours, answer as if you were wearing them.
Please, circle your preferred choice in each question.

A2. Have you noticed that the letters on the screen become blurry while you're working with your computer?

1. None at all 2. Very little 3. Little

4. A moderate amount 5. Much 6. Very much

A22. Have you noticed that, after some time working with your computer, you have to strain to see well?

6. Very much 5. Much 4. A moderate amount

3. Little 2. Very little 1. None at all

A28. While you're reading or writing on your computer, did you feel like you're crossing your eyes?

4. Always 3. Frequently 2. Rarely 1. Never

A30. Have you noticed that, after a lot of time on the computer, the letters appear as a double image?

6. Very much 5. Much 4. A moderate amount

3. Little 2. Very little 1. None at all

A33. Have you noticed that the lights bother you after some time on the computer?

1. Never 2. Almost never 3. A few times

4. Several times 5. Often 6. Very Often

To finish, please indicate to what extent you consider true or false each one of the following statements. If you normally wear glasses or contact lenses during most of your working hours, answer as if you were wearing them.

C21. After some time at the computer, I have to strain to see well

4. Completely true 3. Quite true

1. Quite false 2. Completely false

C24. After some time at the computer, the lights bother me

1. Quite false 2. Completely false

3. Quite true 4. Completely true
