# Supplementary material for: Five levels of performance and two subscales identified in the computer-vision symptom scale (CVSS17) by Rasch, factor, and discriminant analysis
Source: PLoS One. 2018 Aug 28;13(8):e0202173. doi: 10.1371/journal.pone.0202173 (PMC6112632; doi:10.1371/journal.pone.0202173)
Supplement: S10 Appendix — (PDF) [file pone.0202173.s010.pdf]

FACULTAD DE ÓPTICA Y OPTOMETRÍA
UNIVERSIDAD COMPLUTENSE DE MADRID
C/ ARCOS DE JALÓN 118 - 28037 MADRID - ESPAÑA

CVSS17

Apellidos, Nombre:____

Edad:___ **Fecha:**_________

LAS PREGUNTAS QUE SIGUEN SE REFIEREN A CÓMO SE HA SENTIDO DURANTE SUS ÚLTIMAS CUATRO SEMANAS DE TRABAJO

Si usa gafas o lentes de contacto habitualmente en su trabajo, por favor responda a todas las preguntas pensando en cómo se siente cuando sí las lleva puestas.

Por favor, marque con una X su opción preferida en cada pregunta

A2. ¿Ha notado que a veces se le emborronan las letras del ordenador mientras trabaja con él?

1. No, nada 2. Sí, muy poco 3. Sí, un poco
4. Sí, moderadamente 5. Sí, mucho 6. Sí, muchísimo

A22. ¿Ha notado que, tras un tiempo con el ordenador, tiene que esforzarse para poder conseguir ver bien?

6. Sí, muchísimo 5. Sí, mucho 4 Sí, moderadamente
3. Sí, un poco 2. Sí, muy poco 1. No, nada

A28. Mientras lee o escribe con su ordenador ¿tiene la sensación de que se ponga bizco? Tensión

4. Constantemente 3. Frecuentemente 2. Raramente 1. Nunca

A30. ¿Ha notado que cuando pasa mucho tiempo con el ordenador llega un momento en que se acaba viendo las letras dobles?

6. Sí, muchísimo 5. Sí, mucho 4. Sí, moderadamente
3. Sí, un poco 2. Sí, muy poco 1. No, nada

A33. ¿Ha notado que tras un tiempo con el ordenador le molesten las luces?

1. Nunca	2. Casi Nunca	3.Unas Pocas Veces
4. Varias Veces	5. Muchas Veces	6. Muchísimas Veces

POR FAVOR, DIGA SI LE PARECE CIERTA O FALSA CADA UNA DE LAS SIGUIENTES FRASES. Si usa gafas o lentes de contacto habitualmente en su trabajo, por favor responda a todas las preguntas pensando en cómo se siente cuando si los lleva puestos. (Marque con una X)

C21. Tras un tiempo con el ordenador, noto que tengo que esforzarme para ver bien

4. Totalmente cierta	3. Bastante cierta
2. Bastante falsa	1. Totalmente falsa

C24. Tras un tiempo con el ordenador, me molestan las luces

1. Bastante falsa	2. Totalmente falsa
3. Bastante cierta	4. Totalmente cierta
