# Supplementary material for: Five levels of performance and two subscales identified in the computer-vision symptom scale (CVSS17) by Rasch, factor, and discriminant analysis
Source: PLoS One. 2018 Aug 28;13(8):e0202173. doi: 10.1371/journal.pone.0202173 (PMC6112632; doi:10.1371/journal.pone.0202173)
Supplement: S11 Appendix — (PDF) [file pone.0202173.s011.pdf]

| | Response Option | | | | | | |
|---|---|---|---|---|---|---|---|
| **Ítem Id.** | ***1*** | ***2*** | ***3*** | ***4*** | ***5*** | ***6*** | ***7*** |
| ***A2*** | 1 | 1 | 2 | 2 | 3 | 3 | |
| ***A22*** | 1 | 1 | 2 | 2 | 3 | 3 | |
| ***A28*** | 1 | 2 | 3 | 3 | | | |
| ***A30*** | 1 | 1 | 1 | 2 | 2 | 2 | |
| ***A33*** | 1 | 2 | 2 | 3 | 3 | 3 | |
| ***C21*** | 1 | 1 | 2 | 3 | | | |
| ***C24*** | 1 | 1 | 2 | 3 | | | |

**CVSS17 Score = [ (Sum of scores) x 17] / (number of valid responses)]**
